# Epidemiology, healthcare utilization, and related costs among patients with IPF: results from a German claims database analysis

**DOI:** 10.1186/s12931-022-01976-0

**Published:** 2022-03-19

**Authors:** Michael Kreuter, Nils Picker, Larissa Schwarzkopf, Severin Baumann, Agustin Cerani, Roelien Postema, Ulf Maywald, Axel Dittmar, Jonathan Langley, Haridarshan Patel

**Affiliations:** 1 Center for Interstitial and Rare Lung Diseases, Pneumology and Respiratory Critical Care Medicine, Thoraxklinik, University of Heidelberg, Röntgenstrasse 1, 69126 Heidelberg, Germany; 2 German Center for Lung Research, Im Neuenheimer Feld 420, 69120 Heidelberg, Germany; 3 Ingress-Health HWM GmbH, Alter Holzhafen 19, 23966 Wismar, Germany; 4 IFT Institut fuer Therapieforschung, Leopoldstrasse 175, 80804 Munich, Germany; 5 Institute of Health Economics and Health Care Management, Helmholtz Zentrum München (GmbH), German Research Center for Environmental Health, Ingolstaedter Landstrasse 1, 85764 Neuherberg, Germany; 6 Galapagos NV, Generaal De Wittelaan L11 A3, 2800 Mechelen, Belgium; 7 AOK PLUS, Sternplatz 7, 01067 Dresden, Germany; 8 Institut für Pharmakoökonomie und Arzneimittellogistik (IPAM), University of Wismar, Alter Holzhafen 19, 23966 Wismar, Germany

**Keywords:** Idiopathic pulmonary fibrosis, Interstitial lung disease, Incidence, Prevalence, Epidemiology, Healthcare resource utilization, Healthcare costs, Claims data

## Abstract

**Background:**

Idiopathic pulmonary fibrosis (IPF) is a progressive form of fibrosing interstitial pneumonia with poor survival. This study provides insight into the epidemiology, cost, and disease course of IPF in Germany.

**Methods:**

A cohort of incident patients with IPF (n = 1737) was identified from German claims data (2014–2019). Incidence and prevalence rates were calculated and adjusted for age differences compared with the overall German population. All-cause and IPF-related healthcare resource utilization as well as associated costs were evaluated per observed person-year (PY) following the initial IPF diagnosis. Finally, Kaplan–Meier analyses were performed to assess time from initial diagnosis to disease deterioration (using three proxy measures: non-elective hospitalization, IPF-related hospitalization, long-term oxygen therapy [LTOT]); antifibrotic therapy initiation; and all-cause death.

**Results:**

The cumulative incidence of IPF was estimated at 10.7 per 100,000 individuals in 2016, 10.9 in 2017, 10.5 in 2018, and 9.6 in 2019. The point prevalence rates per 100,000 individuals for the respective years were 21.7, 23.5, 24.1, and 24.1. On average, ≥ 14 physician visits and nearly two hospitalizations per PY were observed after the initial IPF diagnosis. Of total all-cause direct costs (€15,721/PY), 55.7% (€8754/PY) were due to hospitalizations and 29.1% (€4572/PY) were due to medication. Medication accounted for 49.4% (€1470/PY) and hospitalizations for 34.8% (€1034/PY) of total IPF-related direct costs (€2973/PY). Within 2 years of the initial IPF diagnosis (23.6 months), 25% of patients died. Within 5 years of diagnosis, 53.1% of patients had initiated LTOT; only 11.6% were treated with antifibrotic agents. The median time from the initial diagnosis to the first non-elective hospitalization was 5.5 months.

**Conclusion:**

The incidence and prevalence of IPF in Germany are at the higher end of the range reported in the literature. The main driver for all-cause cost was hospitalization. IPF-related costs were mainly driven by medication, with antifibrotic agents accounting for around one-third of the total medication costs even if not frequently prescribed. Most patients with IPF do not receive pharmacological treatment, highlighting the existing unmet medical need for effective and well-tolerated therapies.

**Supplementary Information:**

The online version contains supplementary material available at 10.1186/s12931-022-01976-0.

## Background

Idiopathic pulmonary fibrosis (IPF) is a rare, fibrosing interstitial lung disease (ILD) characterized by progressive loss of lung function, dyspnea, and deteriorating quality of life [1, 2]. Most commonly, patients with IPF are male and aged > 60 years at first presentation [3]. The prognosis for patients with IPF is poor, with median survival estimates of 2 to 5 years following diagnosis [4, 5]. Estimating the precise incidence and prevalence of IPF is challenging, given the limited availability of epidemiological data and lengthy diagnostic process.

Although a standardized, internationally accepted diagnostic pathway for IPF exists [3], misdiagnosis as well as delayed diagnosis and treatment often occur [4, 6]. Despite the recent approval of two antifibrotic drugs, pirfenidone and nintedanib, which delay disease progression and may be associated with improved survival [7], prognosis remains poor [3, 8, 9].

Currently, data on the epidemiology and health economic burden of IPF in Germany are sparse. Wälscher et al. (2020) showed that over a 5-year observation period, more than four out of five ILD patients were hospitalized, with Frank et al. (2019) reporting hospitalizations to be the main driver for both total and ILD-associated costs [10, 11]. However, both studies used data collected between 2009 and 2014, and neither focused specifically on IPF.

Against this background, the current claims data study provides greater insight into the epidemiology, healthcare resource utilization (HCRU) and cost, and disease course of IPF in Germany. It describes the demographic and clinical characteristics of incident patients with IPF in Germany and reports current incidence, prevalence, and all-cause mortality rates. Furthermore, this study maps HCRU (including antifibrotic and non-antifibrotic therapy) and provides a detailed description of the associated direct costs. Finally, using proxies for disease deterioration, IPF disease progression is assessed.

## Methods

### Data source and study population

A retrospective analysis was performed using anonymized patient-level insurance claims data from 2014 to 2019, provided by the German regional healthcare provider AOK PLUS. Use of this anonymized data did not require patient informed consent nor research ethics committee approval [12]. This dataset covers approximately 3.4 million individuals from the German federal states of Saxony and Thuringia, corresponding to around 4% of the German population.

**Fig. 1:**
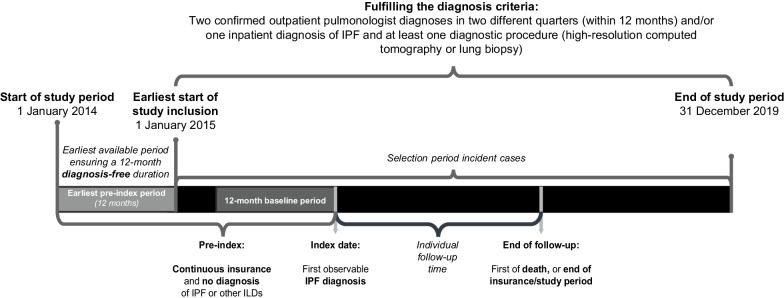
Study period and sample eligibility. *ILD* interstitial lung disease, *IPF* idiopathic pulmonary fibrosis

The sample population was identified using the German Modification of the International Classification of Diseases (ICD-10-GM) code J84.1 for interstitial pulmonary diseases with fibrosis. This approach has previously been used as part of the definition of IPF and ILD-related diseases in German and European claims settings, as well as the hospital discharge setting [10, 13–16]. Individuals were defined as IPF prevalent if ≥ 1 primary inpatient diagnosis of IPF or ≥ 2 outpatient diagnoses of IPF, made by a pulmonologist in two different quarters within 12 months, were observed. In German claims data, there are three types of inpatient diagnoses: main, primary, and secondary. Although there can be multiple primary and secondary diagnoses, only one of the primary diagnoses is defined as the main diagnosis. Cases fulfilling the diagnosis selection criteria were required to be continuously insured (i.e., no discontinuation greater than 30 days) from 1 January 2014 until the first observed IPF diagnosis (index date; Fig. 1). Individuals were excluded from the analysis if they were < 40 years old at the time of IPF diagnosis or did not receive any diagnostic procedure as recommended by clinical guidelines [3, 17], i.e., high-resolution computed tomography (Einheitlicher Bewertungsmassstab [EBM] [18] code 34330) or lung biopsy (Operationen-und Prozedurenschlüssel [OPS] [19] codes 1–430 and 1–581), over the entire study period, or were previously diagnosed with an ICD-10-GM code indicating ILDs other than IPF (Additional file 1: Table S1).

Patients were identified as incident if there was no previous IPF diagnosis in the pre-index period of at least 12 months. Incident patients were followed from the index date to the date of death, loss to follow-up due to end of insurance membership at AOK PLUS, or end of study (31 December 2019; Fig. 1).

### Study variables and statistical analysis

#### Patient baseline characteristics

Baseline characteristics were analyzed descriptively, using summary statistics for continuous variables and frequency statistics for categorical variables. These were based on the index date or a 12-month baseline period before the index date. These include sociodemographic characteristics (age, sex, insurance status), HCRU (outpatient and inpatient), comorbidities, frequently diagnosed diseases, and frequently prescribed drug classes. HCRU comprised frequency of outpatient visits (general practitioner [GP], pulmonologist, other specialists) and hospitalizations, as well as average length of hospital stay (days), all measured in the baseline period. Comorbidities were identified based on ICD-10-GM codes (any diagnosis from inpatient or outpatient settings) and comprised frequencies of patients who had ≥ 1 previous diagnosis of lung cancer, chronic obstructive pulmonary disease (COPD), asthma, hypertension, diabetes mellitus type 2, gastroesophageal reflux disease (GERD), chronic ischemic heart disease, disorders of lipoprotein metabolism and other lipidemia, or heart failure in the baseline period (for ICD-10-GM codes, see Table 1). Comorbidity status was further described based on the Charlson Comorbidity Index (Additional file 1: Table S2) at the index date. For each of the five most common inpatient main diagnoses (based on ICD-10-GM codes) and most common drug classes prescribed (outpatient prescriptions based on Anatomical Therapeutic Chemical [ATC] codes) in the baseline period, the frequency of patients with ≥ 1 diagnosis or prescription was reported. Finally, the number of patients undergoing lung transplantation in the baseline period was reported (for ICD-10-GM codes and OPS codes, see Additional file 1: Table S3).

**Table 1:** Baseline characteristics of patients with IPF

	Value
Number of patients with IPF	1737
Total follow-up period (patient-years)	3720
Median follow-up in years (IQR)	2.0 (0.9–3.2)
Sociodemographic characteristics at index date	
Male sex, n (%)	1173 (67.5)
Age at index date (years), mean (SD)	72.1 (10.4)
Insurance status at index date, n (%)	
Retired	1489 (85.7)
Employed	143 (8.2)
Unemployed	51 (2.9)
Voluntarily insured	37 (2.1)
Family-insured	17 (1.0)
Comorbidities in baseline period	
CCI score at index date, median (IQR)	4.0 (2.0–6.0)
Lung cancer, n (%)	106 (6.1)
COPD, n (%)	691 (39.8)
Asthma, n (%)	282 (16.2)
Heart failure, n (%)	622 (35.8)
Arterial hypertension, n (%)	1411 (81.2)
Type 2 diabetes mellitus, n (%)	647 (37.2)
GERD, n (%)	418 (24.1)
Chronic ischemic heart disease, n (%)	667 (38.4)
Dyslipidemia, n (%)	889 (51.2)
Outpatient visits, hospitalizations, and procedures in baseline period	
Number of GP visits per patient, mean (SD)	4.6 (1.8)
Number of pulmonologist visits per patient, mean (SD)	0.9 (1.2)
Number of other specialist visits per patient, mean (SD)	10.1 (6.2)
Number of all-cause hospitalizations per patient, mean (SD)	1.1 (1.8)
Number of days spent in hospital per patient, mean (SD)	8.2 (18.5)
Lung transplant, n (%)	7 (0.4)
Top five prescribed drug classes in baseline period, n (%)	
Proton pump inhibitors	787 (45.3)
Beta blocking agents, selective	774 (44.6)
Statins	641 (36.9)
Sulfonamide-diuretics, plain	590 (34.0)
Pyrazalones	518 (29.8)
Top five inpatient main diagnoses in baseline period, n (%)	
Pneumonia	102 (5.9)
Heart failure	90 (5.2)
COPD	65 (3.7)
Neoplasm of uncertain behavior of middle ear and respiratory and intrathoracic organs	41 (2.4)
Chronic ischemic heart disease	36 (2.1)

The respective baseline period is 12 months prior to the index date. “Retired” includes pension applicants; For the respective ATC, OPS, and ICD-10-GM codes, see Additional file 1: Table S3

*ATC* Anatomical Therapeutic Chemical, *CCI* Charlson Comorbidity Index, *COPD* chronic obstructive pulmonary disease, *GERD* gastroesophageal reflux disease, *GP* general practitioner, *ICD-10-GM* German Modification of the International Classification of Diseases, *IPF* idiopathic pulmonary fibrosis, *IQR* interquartile range, *OPS* Operationen-und Prozedurenschlüssel/operation and procedure classification, *SD* standard deviation

#### Incidence and prevalence

The point prevalence was assessed on 1 January for each calendar year (2016–2019). The denominator for the point prevalence estimate was the number of individuals aged ≥ 40 years on 1 January for the respective calendar year (2016–2019), who were insured by AOK PLUS on that day and during the preceding 12 months. The numerator was the number of patients in the same age group with ≥ 1 primary inpatient diagnosis of IPF, or ≥ 2 outpatient diagnoses of IPF, made by a pulmonologist in two different quarters within the preceding 12 months. Patients needed to be alive on 1 January of the respective calendar year.

The yearly cumulative incidence was calculated by dividing the number of newly diagnosed patients with IPF in each calendar year by the number of subjects at risk at the beginning of the same year. At-risk subjects had continuous insurance at AOK PLUS and no confirmed IPF (as described above) in the preceding year. At-risk subjects were required to be ≥ 40 years at the beginning of the calendar year. The numerator was the number of patients in the same age group who had ≥ 1 primary inpatient diagnosis of IPF, or ≥ 2 outpatient diagnoses of IPF, made by a pulmonologist in two different quarters within the respective calendar year.

Both the cumulative incidence rates and point prevalence rates were standardized according to the age distribution of the German statutory health insurance (SHI) population [20].

#### HCRU

Information on all-cause and IPF-related HCRU was collected from the patient individual follow-up period (including index date). For outpatient physician visits, cases were considered IPF-related if any diagnosis was coded as ICD-10-GM J84.1. Hospitalizations and inpatient rehabilitations were defined as IPF-related if the associated main diagnosis was J84.1.

Outcomes for all-cause and IPF-related HCRU consisted of the number of hospitalizations, days spent in hospital, number of outpatient visits, inpatient rehabilitation stays, and days in inpatient rehabilitation, all per person-year (PY). Frequency of patients with at least one of the following was reported: hospitalization, GP visit, pulmonologist visit, and visit to another specialist. During the follow-up period, the frequency of patients with ≥ 1 prescription for systemic corticosteroids (ATC code: H02), inhaled corticosteroids (R03BA), *N*-acetylcysteine (R05CB01), azathioprine (L04AX01), nintedanib (L01XE31), or pirfenidone (L04AX05) was reported. For each antifibrotic agent, the frequency of prescriptions per PY was also reported. Time from initial IPF diagnosis to the start of antifibrotic therapy was analyzed using the Kaplan–Meier method.

#### Costs

All-cause costs included costs for inpatient care (i.e., hospitalizations), outpatient care, medication, medical aids, and remedies, as well as inpatient rehabilitation stays. The same definitions for IPF-related outpatient visits, hospitalizations and rehabilitation stays as above were used to estimate the costs associated with these HCRU variables. IPF-related medication costs consisted exclusively of costs associated with outpatient prescriptions of nintedanib and pirfenidone. IPF-related costs for medical aids and remedies consisted of costs associated with inhalation and respiration devices included in product group 14 of the medical aids directory (“Hilfsmittelverzeichnis”) [21], published by the National Association of Statutory Health Insurance Funds (“GKV-Spitzenverband”). They also consisted of remedies associated with respiratory disorders in the remedies catalog (“Heilmittelkatalog”) [22], published by the National Association of Statutory Health Insurance Physicians (“Kassenärztliche Bundesvereinigung”) (e.g., physiotherapy, breathing therapy, connective tissue massage). Costs related to inpatient care claims, which cover all services and pharmacological treatments during hospitalization stays, are based on the diagnosis-related groups’ reimbursement codes [23]. Reimbursement of outpatient care services in Germany is regulated by the EBM. These services were valued based on predefined weighted points that were multiplied by a uniform orientation value. The values used in this study ranged from €0.1027 in 2015 to €0.1082 in 2019 [24]. Outpatient prescriptions were valued based on the pharmacy retail price (“Apothekenabgabepreis”) at the respective date of prescription [25]. All-cause and IPF-related costs were reported as cost rates per PY for the patient individual follow-up period.

#### All-cause mortality and disease deterioration

Analyses of time to disease deterioration and death were based on the Kaplan–Meier method. The individual follow-up period began with the initial IPF diagnosis. Censoring criteria were loss to follow-up; end of the study period; or, in the case of disease deterioration, death.

As cause of death is not documented within claims data we refer to all-cause instead of IPF-related mortality.

German claims data contain no information on disease deterioration as measured by clinical/lung function parameters. Therefore, information on disease deterioration was obtained using the proxies of non-elective hospitalizations [26], IPF-related hospitalizations, and long-term oxygen therapy (LTOT) initiation [27]. Hospitalizations were defined as IPF-related if they were denoted with the main diagnosis (ICD-10-GM codes) of interstitial pulmonary diseases with fibrosis (J84.1), respiratory failure (J96), respiratory infections (J09–J22, J40), pneumothorax (J93), pulmonary embolism (I26), or pulmonary hypertension (I27) [11]. LTOT was considered to start on the date of the first documented LTOT-related OPS code (Additional file 1: Table S4) or prescription of product group 14 of the medical aids directory. A composite endpoint based on all three deterioration proxies was also analyzed.

All statistical analyses were conducted using Stata (version 16.1), Microsoft Excel (version 2105), and MySQL (SQL Server 2019).

## Results

### Patient baseline characteristics

**Fig. 2:**
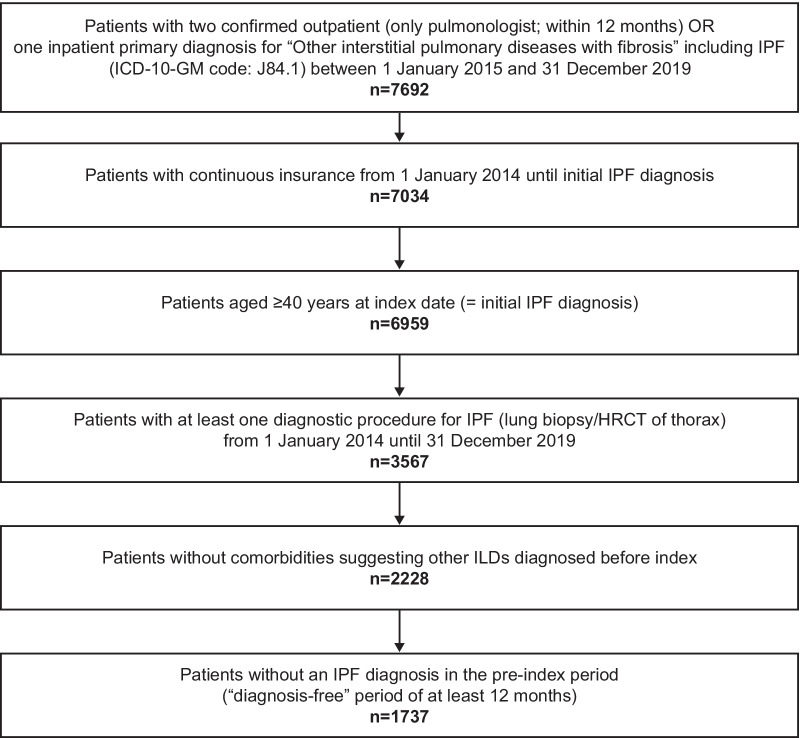
Attrition chart. *HRCT* high-resolution computed tomography, *ICD-10-GM* German Modification of the International Classification of Diseases, *ILD* interstitial lung disease, *IPF* idiopathic pulmonary fibrosis

Based on the predefined selection criteria, 1737 incident patients with IPF were identified (Fig. 2), with a median follow-up time per patient of 2.0 years.

Mean age at index date was 72.1 years, corresponding to a high proportion of retired patients (85.7%); 67.5% of patients were male (Table 1). The most common reasons for inpatient stays in the baseline period were pulmonary and heart diseases. In the baseline year, 81.2% of newly diagnosed patients with IPF had concomitant arterial hypertension. Other common comorbidities were dyslipidemia (51.2%), COPD (39.8%), chronic ischemic heart disease (38.4%), heart failure (35.8%), and type 2 diabetes mellitus (37.2%). A large number of patients were treated with proton pump inhibitors (45.3%), or drugs for the prevention of cardiovascular disease, such as beta blocking agents (44.6%) and statins (36.9%; Table 1).

### Incidence and prevalence

**Fig. 3:**
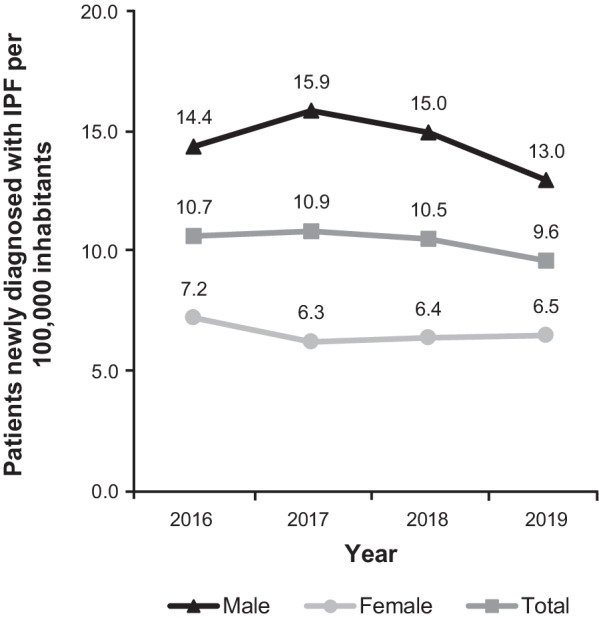
Cumulative incidence of IPF (2016–2019). Incidence rates from Saxony/Thuringia were adjusted for age differences compared with the German statutory health insurance population. *IPF* idiopathic pulmonary fibrosis

After adjusting for age differences compared with the German SHI population, the following cumulative IPF incidence rates per 100,000 individuals were calculated: 10.7 in 2016, 10.9 in 2017, 10.5 in 2018, and 9.6 in 2019. The female IPF incidence rates for these years were 7.2, 6.3, 6.4, and 6.5, and the male rates were 14.4, 15.9, 15.0, and 13.0, respectively (Fig. 3).

**Fig. 4:**
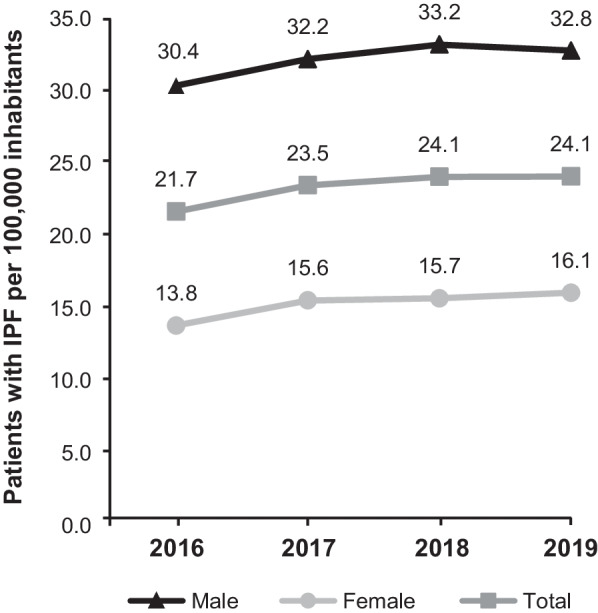
Point prevalence of IPF (2016–2019). Prevalence rates from Saxony/Thuringia were adjusted for age differences compared with the German statutory health insurance population. *IPF* idiopathic pulmonary fibrosis

The age-adjusted point prevalence (at the start of each calendar year) per 100,000 individuals was 21.7 in 2016, 23.5 in 2017, 24.1 in 2018, and 24.1 in 2019. The female point prevalence rates for the respective years were 13.8, 15.6, 15.7, and 16.1, while the male rates were 30.4, 32.2, 33.2, and 32.8, respectively (Fig. 4).

### HCRU

On average, patients with newly diagnosed IPF visited a physician ≥ 14 times per year (4.4 GP visits, 1.8 pulmonologist visits, 8.2 other specialist visits). Approximately three visits per PY were related to IPF (1.3 GP visits, 1.2 pulmonologist visits, 0.4 other specialist visits). Incident patients with IPF were hospitalized 1.8 times per PY, with IPF as the main diagnosis for one-sixth of hospitalizations. During follow-up, 93.4% (n = 1623) of patients were hospitalized at least once, 37.5% (n = 653) of hospitalizations due to IPF. The number of days spent in hospital was 15.7 per PY, of which an average of 2.0 days were related to IPF. During the follow-up period, 93.7% (n = 1628) of patients visited their GP, 39.1% (n = 680) of which were due to IPF. A high percentage of patients (92.1%) visited other specialists, although fewer visits were IPF-related (26.5%). By comparison, the percentage of patients visiting a pulmonologist during the follow-up period was lower (67.5%), but a higher proportion of these visits were IPF-related (47.3%) (Table 2).

**Table 2:** HCRU after initial diagnosis for IPF (N = 1737)

	Events per person-year	
	All-cause	IPF-related
Number of hospitalizations	1.8	0.3
Number of hospital days	15.7	2.0
Number of GP visits	4.4	1.3
Number of pulmonologist visits	1.8	1.2
Number of other specialist visits	8.2	0.4
Number of inpatient rehabilitation stays^a^	< 0.1	< 0.1
Number of days in inpatient rehabilitation^a^	1.4	0.1

	No. of patients with ≥ 1 event	
Patients with hospitalization, n (%)	1623 (93.4)	653 (37.5)
Patients with GP visit, n (%)	1628 (93.7)	680 (39.1)
Patients with pulmonologist visit, n (%)	1173 (67.5)	821 (47.3)
Patients with visit to other specialist(s), n (%)	1599 (92.1)	461 (26.5)

Total person time in years: 3720

*GP* general practitioner, *HCRU* healthcare resource utilization, *IPF* idiopathic pulmonary fibrosis

^a^This number does not include rehabilitation of the working population, which is covered by statutory pension insurance; statutory health insurance only supports rehabilitation outside of the workforce

**Fig. 5:**
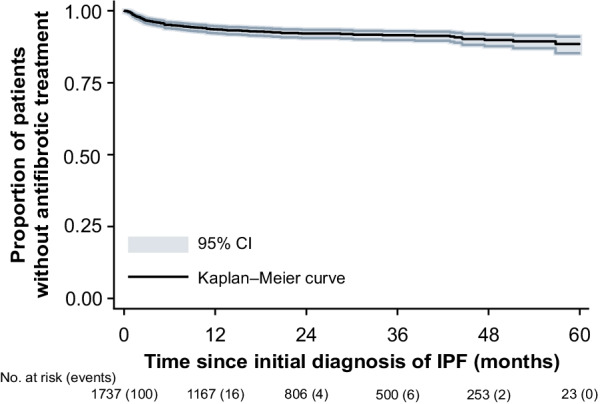
Kaplan–Meier analysis of antifibrotic therapy. Number of events are in brackets. *CI* confidence interval, *IPF* idiopathic pulmonary fibrosis

For time from initial IPF diagnosis to initiation of antifibrotic therapy (Fig. 5), 8.9% (n = 120) of patients had received either nintedanib or pirfenidone within 3 years. At 5 years, 128 (11.6%) patients had received antifibrotic therapy, with 72 (56.3%) of these patients receiving nintedanib alone, 33 (25.8%) pirfenidone alone, and 23 (18.0%) receiving both agents sequentially.^1^ The prescription frequency for nintedanib and pirfenidone was 0.3 and 0.1 per PY, respectively. The small number of treated patients prevented a meaningful analysis of treatment patterns.

At least one prescription for IPF-related, non-antifibrotic agents was received by 51.4% (n = 893) of patients. During follow-up, 42.5% (n = 739) of newly diagnosed patients received systemic corticosteroids, with a lower percentage receiving inhaled corticosteroids (14.5%; n = 251). A small number of patients received acetylcysteine (6.2%; n = 108) or azathioprine (2.2%; n = 39).

A total of 45.1% (n = 783) of patients did not receive the antifibrotic or non-antifibrotic agents discussed above.

### Costs

**Fig. 6:**
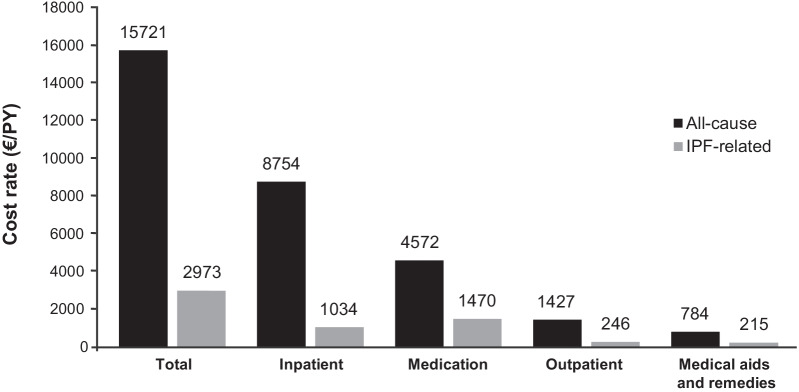
Cost rates (2015–2019). Person time in years: 3720. All measurements in Euro per PY. Inpatient rehabilitation costs were negligible and therefore not shown (all-cause €185/PY, IPF-related €8/PY). *IPF* idiopathic pulmonary fibrosis, *PY* person-year, *€* euro

Total all-cause direct healthcare costs for the entire observation period were €58.5 million, which corresponds to €15,721 per PY. IPF-related direct costs accounted for 18.9% of total direct costs (€11.1 million; €2973/PY). The main cost drivers were hospitalization and medication, accounting for 55.7% (€8754/PY) and 29.1% (€4572/PY) of total all-cause direct costs, respectively. Antifibrotic therapy accounted for 49.4% (€1470/PY) of IPF-related direct costs, with 34.8% (€1034/PY) related to ‘hospitalizations’. Antifibrotic therapy also accounted for 32.1% of total drug costs (Fig. 6).

### All-cause mortality and disease deterioration

**Fig. 7:**
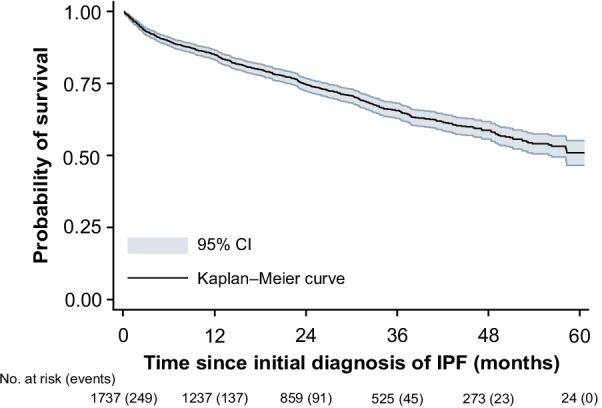
Kaplan–Meier analysis of all-cause mortality. Number of events are in brackets. *CI* confidence interval, *IPF* idiopathic pulmonary fibrosis

In patients with newly diagnosed IPF, 15.1% (n = 249) died in the 1st year following initial diagnosis. The 25th percentile was reached within less than 2 years (23.6 months; 95% confidence interval [CI] 21.3–26.5). Within the 5-year follow-up period, 545 (49.3%) deaths were observed (Fig. 7).

**Fig. 8:**
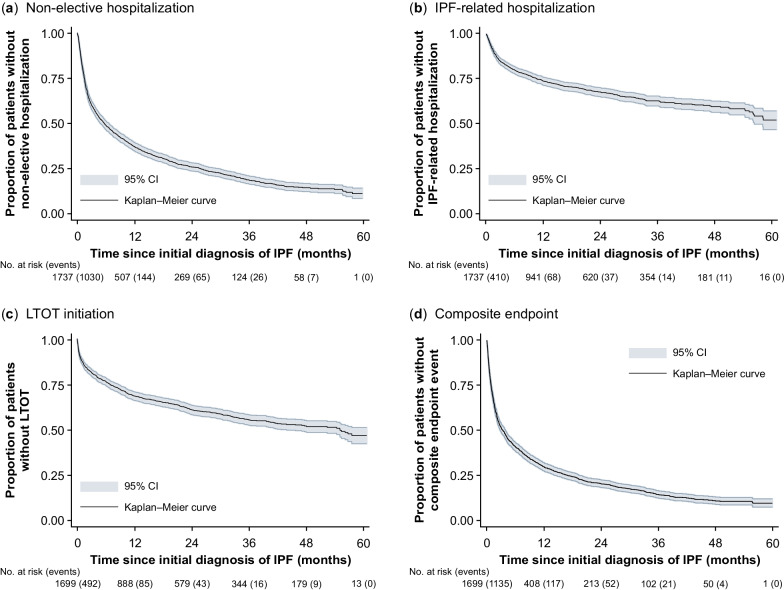
Kaplan–Meier analysis. **a** Non-elective hospitalization, **b** IPF-related hospitalization, **c** LTOT initiation, **d** composite endpoint. Number of events are in brackets. Non-elective hospitalizations were identified based on records for “emergency admission”, with the following admission codes being excluded: “delivery of birth”, “organ removal”, “pre-hospital treatment”, “inpatient stay with prior pre-hospital treatment”. The composite endpoint consists of non-elective hospitalization, IPF-related hospitalization and LTOT initiation. *CI* confidence interval, *IPF* idiopathic pulmonary fibrosis, *LTOT* long-term oxygen therapy

The median time from initial IPF diagnosis to first non-elective hospitalization for any cause was 5.5 months (95% CI 4.7–6.3); 82.0% (n = 1239) of patients experienced a non-elective hospitalization within 3 years. The 1-year probability of non-elective hospitalization was 63.3% (Fig. 8).

Within 10.8 months (95% CI 8.9–13.7), one-quarter of patients were hospitalized due to IPF, increasing to 37.0% (n = 515) within 3 years. Throughout the 5-year follow-up period, 540 patients (47.6%) experienced an IPF-related hospitalization.

Thirty-eight patients were treated with LTOT on the index date and therefore excluded from the LTOT initiation analysis. Within 6.9 months (95% CI 5.8–8.6), one in four patients started LTOT. The median time to LTOT initiation was estimated at 55.1 months (95% CI 43.9–not available). Among the 645 patients who were treated with LTOT during the observable follow-up period (excluding index date), 25.6% (n = 165) had received LTOT in the 12-month baseline period.

The median event-free time for the composite endpoint of non-elective hospitalizations [26], IPF-related hospitalizations, and/or LTOT, was 3.4 months (95% CI 2.8–3.9), with the 75th percentile reached after 17.0 months (95% CI 14.4–19.4).

## Discussion

There is a substantial gap in knowledge relating to the epidemiology, economic burden, and disease course of IPF worldwide. Our retrospective claims data study in Germany aimed to address this gap by describing the demographic and clinical characteristics of patients with IPF, providing updated epidemiological data, assessing disease progression, and estimating the HCRU and costs associated with this disease.

Between 2015 and 2019, 1737 incident IPF patients were identified. Following adjustments for age differences compared with the German SHI population, cumulative incidence was estimated to be between 9.6 and 10.9 and point prevalence between 21.7 and 24.1 per 100,000 individuals for 2016–2019. The point prevalence showed a slight upward trend, whereas the cumulative incidence was stable. There are no current population-based incidence or prevalence rates for IPF in Germany, and substantial variation is reported in data from other countries. In a review of 34 studies from 21 countries published between 1968 and 2012, Hutchinson et al. [28] reported incidence estimates of 3–9 cases per 100,000 individuals per year in Europe and North America. The German Guideline for Diagnosis and Management of IPF from 2013 reported prevalence estimates of 2–29 cases per 100,000 individuals [29]. Compared with these estimates, our data suggest a relatively high incidence and prevalence of IPF in Germany. It should be noted that Saxony and Thuringia are rural compared with other German regions. The current population in these regions may be less affected by air pollution, which is a recognized risk factor for the incidence and acute exacerbation of IPF [30, 31]. However, historical pollution levels in Saxony and Thuringia may have been higher, as both regions were part of the German Democratic Republic, in which pollutant emissions were previously extremely high [32–34]. The socio-economic disparities that still exist between German states may limit how representative these incidence and prevalence rates are for Germany as a whole; however, mortality in most age groups has been shown to converge across the former East–West political divide [35, 36].

Our IPF population was older, predominantly male and demonstrated a high burden of comorbidities [15, 37]. Hypertension, COPD, ischemic heart disease, heart failure, type 2 diabetes, GERD, and asthma, which have all been identified as important IPF comorbidities [1, 10, 37, 38], were prevalent. However, it is common for pulmonary fibrosis to be misdiagnosed as heart disease, COPD, or asthma, which might result in an overestimation of comorbidity numbers [39]. The high proportion of hospitalizations for pulmonary diseases also suggests delayed diagnosis of IPF.

Data on HCRU for patients with IPF in Germany are sparse. Wälscher et al. [10] reported that 86% of incident ILD patients were hospitalized over a 5-year observation period (2009–2014). In our study, for the same follow-up period, a higher percentage of patients with IPF (93%) were hospitalized, consistent with the characterization of IPF as a more serious form of ILD. Within 5 years of the initial IPF diagnosis, approximately one-tenth of patients had been treated with antifibrotic agents in our sample. This is low when compared with other reports. Behr et al. [7] found that around half of German patients with IPF received either nintedanib or pirfenidone. However, Behr et al. [7] used registry data from specialized ILD centers, whereas our claims dataset represents a broader population of patients with IPF, which could explain this difference. Another reason for the low use of pirfenidone and nintedanib in our sample could be the fact that diffusion to routine care takes time and these medications were only approved in Germany in 2012 and 2015, respectively.

Our study highlights the economic burden of IPF, with healthcare costs per patient three times higher than the average yearly healthcare expenditure per insured individual in Germany [40]. Hospitalizations and medication were the main cost drivers, with more than half of the total all-cause direct costs for newly diagnosed patients with IPF caused by hospitalizations and almost one-third by medications. For IPF-related direct costs, medication accounted for around half of total costs and hospitalizations for more than one-third. Frank et al. [11] examined the economic burden of ILDs (IPF and sarcoidosis) and their associated comorbidities using a claims dataset that covered about one-third of the German population. This study looked at an earlier period (2009–2014) and distinguished between all-cause costs and ILD-related costs. The mean annual per capita healthcare costs for patients with IPF were lower than our study (€12,111 vs €15,721), which can partly be explained by the rise in per capita healthcare expenditure and price levels. The study’s findings are consistent with our own, as hospital costs were the main driver of total costs, followed by medication. By contrast, the study from Frank et al. [11] found that medication accounted for only 13.9% of ILD-related costs, compared with 49.4% of IPF-related costs in our study. Although IPF is the most common subtype of fibrosing ILD, a direct comparison of these two studies is not appropriate. However, some of the differences may result from the earlier time period examined by Frank et al. [11], as pirfenidone and nintedanib only became available in 2012 and 2015, respectively. Over the last decade, there has been an increase in the cost of antifibrotic agent prescriptions in Europe due to these licenses [8].

We report a 5-year survival probability of around 50%, whereas a Swedish study using similar inclusion criteria for IPF (ICD-10 code of J84.1, aged ≥ 40 years) reported a rate of approximately 30% over the same duration, and a median survival time of 2.6 years [15]. Our results are consistent with an Australian IPF registry data study, which estimated the cumulative mortality rate 1, 2, 3, and 4 year(s) after diagnosis at 5%, 24%, 37%, and 44% (vs 15%, 26%, 35%, and 41% in our study), respectively [38]. Using German registry data, Behr et al. [7] reported the 1- and 2-year mortality rates for patients with or without antifibrotic therapy as 13% vs 54% and 38% vs 79%, respectively. Global estimates for median survival are approximately 2–5 years [4, 5], indicating a comparatively high overall survival in our sample.

In line with historical international guidelines for the management of patients with IPF [17], a high percentage of patients using LTOT after initial diagnosis were identified, with the 25th percentile reached after 6.9 months and the median after 55.1 months. To our knowledge, there are no studies analyzing LTOT use among patients with IPF using time-to-event analysis. Recent German studies based on registry data reported LTOT use of 33.1% and 32.3% among patients with IPF, with a mean disease duration of 2.3 and 2.0 years, respectively [1, 41].

Our study suggests a relatively rapid worsening of health in newly diagnosed patients with IPF. A US claims data study from 2016 found an all-cause hospitalization risk of 39% within 1 year of IPF diagnosis, with a US registry study from 2020 reporting a 30% hospitalization risk for the same period [42, 43]. By contrast, the 1-year probability of all-cause, non-elective hospitalization was 63% in our sample. Hospitalization rates for ILD patients in Germany have been reported to be higher than those for patients with IPF in the US [10]. This could be explained by the different inclusion criteria used by Wälscher et al. [10], and different referral and hospitalization patterns between the US and Europe. These authors also used German claims data, reporting a median time from initial IPF diagnosis to the first ILD-related hospitalization of around 15 months [10], whereas in our analysis the median time from initial IPF diagnosis to the first IPF-related hospitalization was not reached after 5 years. This could be explained by the use of a wider list of ILD codes used by Wälscher et al. [10] to define ILD.

### Limitations

A number of limitations, some of which are inherent to retrospective claims database analyses, were identified in the current study. This includes the information contained in the AOK PLUS dataset. Direct measures of functional status, such as forced expiratory volume in one second, forced vital capacity, and 6-min walk test, are not included. As a result, proxies were used to describe disease deterioration. German claims data do not report specific clinical reasons for hospitalizations, containing only ICD-10 codes. Inpatient rehabilitation is only partially covered in German claims data, as SHI only supports rehabilitation outside of the workforce, with rehabilitation of the working population covered by statutory pension insurance. As such, the number of rehabilitation days and associated costs in this study are likely to be an underestimation. Considering the mean age of patients with IPF in our sample, we do not believe this bias to be large. A further drawback of German claims data is the lack of information on drugs dispensed from hospital pharmacies, as well as nursing and sick leave costs; these latter two add to the indirect economic burden of disease.

There are also limitations with respect to how information is reported in the AOK PLUS dataset. For example, outpatient diagnoses and costs are not linked to single visits but are reported by individual physicians per quarter. The total number of IPF-related visits and related costs may therefore be misestimated, if invoiced by the same physician. A drawback in identifying IPF patients via the ICD-GM-10 code J84.1, is that this code includes a range of ILDs such as diffuse pulmonary fibrosis, fibrosing alveolitis (cryptogenic) (both formerly used instead of IPF), and Hamman-Rich syndrome. The code can also include other idiopathic interstitial pneumonias such as nonspecific interstitial pneumonia. A proportion of the IPF cases identified by this study might therefore potentially be misclassified, which could be a reason for the relatively high prevalence and incidence numbers reported, compared with other studies outside of Germany. However, as IPF is the most common fibrosing ILD, possible misclassification of the case definition is reduced [8]. The risk of possible misclassification was further reduced by excluding patients with diagnoses suggestive of ILDs other than IPF (Additional file 1: Table S1). Furthermore, it is not possible to unambiguously disentangle the individual contribution of distinct, potentially life-limiting diseases in the multimorbid patient to the outcomes observed. In this context, overlapping, interacting symptoms and complications of IPF and co-existing diseases (e.g., cardiovascular diseases) are assumed to detrimentally affect assignment to as well as rates, timing and costs of IPF-related hospitalizations. This is an unsolved methodological issue for any diagnosis-based uncontrolled study.

Finally, some limitations are unrelated to the dataset. Prescriptions for both drugs containing nintedanib in Germany (Ofev and Vargatef) were included to estimate the prevalence of antifibrotic therapy as a treatment against IPF; however, Vargatef is only licensed for lung cancer. Our sample contains patients diagnosed with both IPF and lung cancer, yet the rationale for a specific prescription is unknown. As such, Vargatef was considered to be antifibrotic therapy in this analysis.

## Conclusions

The incidence and prevalence of IPF in Germany are at the higher end of the range reported in the literature, with the point prevalence slightly increasing between 2016 and 2019. The overall survival of newly diagnosed IPF patients was relatively high in our sample. The main all-cause cost driver for patients with IPF was hospitalization. By contrast, IPF-related costs were mainly driven by medication, with antifibrotic agents accounting for around one-third of the total medication costs, even if not frequently prescribed. Physicians appear hesitant to prescribe antifibrotic therapy, whereas LTOT was initiated more frequently. Despite the availability of antifibrotic therapies, most patients with IPF do not receive pharmacological treatment, highlighting the existing unmet medical need for effective and well-tolerated IPF-targeted therapies.

## Supplementary Information

**Additional file 1: Table S1.** ICD-10-GM codes for study exclusion. **Table S2.** Charlson Comorbidity Index. **Table S3.** ATC/OPS/ICD-10-GM codes for baseline characteristics. **Table S4.** OPS codes for disease deterioration proxies.

## Data Availability

The data that support the findings of this study are available from AOK PLUS; however, restrictions apply to the availability of these data, which were used under license for the current study and are not publicly available. Data are, however, available from the authors upon reasonable request and with permission from AOK PLUS.

## Abbreviations

ATC: Anatomical Therapeutic Chemical
CCI: Charlson Comorbidity Index
CI: Confidence interval
COPD: Chronic obstructive pulmonary disease
EBM: Einheitlicher Bewertungsmassstab/Uniform evaluation standard
GERD: Gastroesophageal reflux disease
GP: General practitioner
HCRU: Healthcare resource utilization
HRCT: High-resolution computed tomography
ICD-10-GM: German Modification of the International Classification of Diseases
ILD: Interstitial lung disease
IPF: Idiopathic pulmonary fibrosis
IQR: Interquartile range
LTOT: Long-term oxygen therapy
OPS: Operationen-und Prozedurenschlüssel/operation and procedure classification
PY: Person-year
SD: Standard deviation
SHI: Statutory health insurance

## Footnotes

1 Nintedanib is sold under the brand names Vargatef and Ofev, the latter of which accounted for 97.1% of prescriptions in our sample.
