# Supplementary material for: Epidemiology, healthcare utilization, and related costs among patients with IPF: results from a German claims database analysis
Source: Respir Res. 2022 Mar 19;23:62. doi: 10.1186/s12931-022-01976-0 (PMC8933882; doi:10.1186/s12931-022-01976-0)
Supplement: Supplementary file 1 — Additional file 1: Table S1. ICD-10-GM codes for study exclusion. Table S2. Charlson Comorbidity Index. Table S3. ATC/OPS/ICD-10-GM codes for baseline characteristics. Table S4. OPS codes for disease deterioration proxies. [file 12931_2022_1976_MOESM1_ESM.docx]

# Additional information

**Table S1** ICD-10-GM codes for study exclusion

| **ICD-10-GM code** | **Description** |
| --- | --- |
| D86 | Sarcoidosis |
| J60 | Coal worker pneumoconiosis |
| J61 | Pneumoconiosis due to asbestos and other mineral fibers |
| J62 | Pneumoconiosis due to dust containing silica |
| J63 | Pneumoconiosis due to other inorganic dusts |
| J64 | Unspecified pneumoconiosis |
| J66 | Airway disease due to specific organic dust |
| J67 | Allergic alveolitis and hypersensitivity pneumonitis due to organic dust |
| J68 | Respiratory conditions due to inhalation of chemicals, gases, fumes, and vapours |
| J70 | Respiratory conditions due to other external agents |
| J82 | Pulmonary eosinophilia, not elsewhere classified |
| J84.0 | Alveolar and parietoalveolar conditions |
| J84.8 | Other specified interstitial pulmonary diseases |
| J99.1 | Respiratory disorders in other diffuse connective tissue disorders |
| K50 | Crohn’s disease |
| M06 | Other rheumatoid arthritis |
| M31 | Other necrotizing vasculopathies |
| M32 | Systemic lupus erythematosus |
| M33 | Dermatopolymyositis |
| M34 | Systemic sclerosis |
| M35 | Other systemic involvement of connective tissue |
| M45 | Ankylosing spondylitis |

ICD-10-GM: German Modification of the International Classification of Diseases

**Table S2** Charlson Comorbidity Index

| **No** | **Comorbidity** | **Charlson score** | **ICD-10-GM code** |
| --- | --- | --- | --- |
| 1 | Coronary artery disease | 1 | I20.-, I21.-, I22.-, I23.-, I24.-, I25.- |
| 2 | Congestive heart failure | 1 | I11.-, I50.- |
| 3 | Peripheral vascular disease | 1 | I73.-, I74.-, I77.- |
| 4 | Cerebrovascular disease | 1 | G45.-, G46.-, I6.- |
| 5 | Dementia | 1 | F00.-, F01.-, F02.-, F03.-, G30.- |
| 6 | Chronic pulmonary disease | 1 | J4.-, J6.- w/o J67.-, J68.-, J69.- |
| 7 | Connective tissue disorder | 1 | M05.-, M06.-, M07.-, M08.-, M3.- |
| 8 | Peptic ulcer disease | 1 | K25.-, K26.-, K27.-, K28.- |
| 9 | Mild liver disease | 1 | B18.-, K70.-, K73.-, K75.- |
| 10 | Diabetes mellitus without complications | 1 | E109.-, E119.-, E129.-, E139.-, E149.- |
| 11 | Hemiplegia | 2 | G81.-, G82.- |
| 12 | Moderate or severe renal disease | 2 | N17.-, N18.-, N19.- |
| 13 | Diabetes mellitus with end-organ damage | 2 | E10.-, E11.-, E12.-, E13.-, E14.- |
| 14 | Tumor without metastases, leukemia, lymphoma, multiple myeloma | 2 | C |
| 15 | Moderate or severe liver disease | 3 | K72.-, K74.-, I85.- |
| 16 | Metastatic solid tumor | 6 | C77.-, C78.-, C79.-, C80.- |
| 17 | AIDS | 6 | B20.-, B21.-, B22.-, B23.-, B24.- |
| 18 | Age factor (excluded from the index) | For each decade ≥50 years of age, 1 point was added to the score | |

ICD-10-GM: German Modification of the International Classification of Diseases

**Table S3** ATC/OPS/ICD-10-GM codes for baseline characteristics

| **Variable** | **Code** | **Coding system** |
| --- | --- | --- |
| Lung caner | C34 | ICD-10-GM |
| COPD | J44 | ICD-10-GM |
| Asthma | J45 | ICD-10-GM |
| Heart failure | I50 | ICD-10-GM |
| Arterial hypertension | I10 | ICD-10-GM |
| Type 2 diabetes mellitus | E11 | ICD-10-GM |
| GERD | K21 | ICD-10-GM |
| Chronic ischemic heart disease | I25 | ICD-10-GM |
| Dyslipidemia | E78 | ICD-10-GM |
| Pneumonia | J18 | ICD-10-GM |
| Neoplasm of uncertain behavior of middle ear and respiratory and intrathoracic organs | D38 | ICD-10-GM |
| Lung transplant | 5-335 | OPS |
|  | Z94.2 | ICD-10-GM |
|  | Z94.3 | ICD-10-GM |
|  | T86.8 | ICD-10-GM |
|  | T86.3 | ICD-10-GM |
| Proton pump inhibitors | A02BC | ATC |
| Beta blocking agents, selective | C07AB | ATC |
| Statins | C10AA | ATC |
| Sulfonamide-diuretics, plain | C03CA | ATC |
| Pyrazalones | N02BB | ATC |

ATC: Anatomical Therapeutic Chemical; COPD: chronic obstructive pulmonary disease; GERD: gastroesophageal reflux disease; ICD-10-GM: German Modification of the International Classification of Diseases; OPS: Operationen-und Prozedurenschlüssel/operation and procedure classification

**Table S4** OPS codes for disease deterioration proxies

| **OPS code** | **Description** | **Disease deterioration proxy** |
| --- | --- | --- |
| 8-713 | Machine ventilation and respiratory support for adults | LTOT |
| 8-714 | Special procedure for machine ventilation in severe respiratory failure | LTOT |
| 8-716 | Setting of a home mechanical ventilation | LTOT |

LTOT: long-term oxygen therapy; OPS: Operationen-und Prozedurenschlüssel/operation and procedure classification
